# HLA-A is a Predictor of Hepatitis B e Antigen Status in HIV-Positive African Adults

**DOI:** 10.1093/infdis/jiv592

**Published:** 2015-12-09

**Authors:** Philippa C. Matthews, Jonathan M. Carlson, Apostolos Beloukas, Amna Malik, Pieter Jooste, Anthony Ogwu, Roger Shapiro, Lynn Riddell, Fabian Chen, Graz Luzzi, Gerald Jesuthasan, Katie Jeffery, Nebojsa Jojic, Thumbi Ndung'u, Mary Carrington, Philip J. R. Goulder, Anna Maria Geretti, Paul Klenerman

**Affiliations:** 1 Nuffield Department of Medicine; 2 Department of Paediatrics, University of Oxford; 3 Department of Infectious Diseases and Microbiology, Oxford University Hospitals NHS Trust; 4 NIHR Biomedical Research Centre, John Radcliffe Hospital, Oxford; 5 Department of Clinical Infection, Microbiology and Immunology, Institute of Infection and Global Health, University of Liverpool; 6 Integrated Sexual Health Services, Northampton General Hospital, Cliftonville; 7 Department of Sexual Health, Royal Berkshire Hospital, Reading; 8 Department of Sexual Health, High Wycombe Hospital, Buckinghamshire, United Kingdom; 9 Microsoft Research, eScience Group, Redmond, Washington; 10 Department of Immunology and Infectious Diseases, Harvard School of Public Health, Boston; 11 The Ragon Institute of MGH, MIT and Harvard, Cambridge, Massachusetts; 12 Cancer and Inflammation Program, Laboratory of Experimental Immunology, Leidos Biomedical Research, Frederick National Laboratory for Cancer Research, Maryland; 13 Paediatric Department, Kimberley Hospital, Northern Cape; 14 HIV Pathogenesis Programme, Doris Duke Medical Research Institute, Nelson R. Mandela School of Medicine; 15 KwaZulu-Natal Research Institute for Tuberculosis and HIV, University of KwaZulu-Natal, South Africa; 16 Botswana Harvard AIDS Institute Partnership, Gaborone, Botswana; 17 Max Planck Institute for Infection Biology, Berlin, Germany

**Keywords:** HBV, HIV, coinfection, immunology, CD8^+^ T cells, HLA, HBeAg (hepatitis B e antigen), viral hepatitis, Africa

## Abstract

Outcomes of chronic infection with hepatitis B virus (HBV) are varied, with increased morbidity reported in the context of human immunodeficiency virus (HIV) coinfection. The factors driving different outcomes are not well understood, but there is increasing interest in an HLA class I effect. We therefore studied the influence of HLA class I on HBV in an African HIV-positive cohort. We demonstrated that virologic markers of HBV disease activity (hepatitis B e antigen status or HBV DNA level) are associated with HLA-A genotype. This finding supports the role of the CD8^+^ T-cell response in HBV control, and potentially informs future therapeutic T-cell vaccine strategies.

Genome-wide studies of hepatitis B virus (HBV) infection have primarily identified HLA class II as a determinant of viral clearance, disease status, and response to therapy [1, 2]. Nevertheless, a small but growing body of evidence suggests a role for HLA class I in modulating outcomes of chronic HBV infection [1, 3, 4], underpinning the hypothesis that CD8^+^ T-cell responses could contribute to HBV immune containment [5]. Among HLA class I alleles, a locus-specific hierarchy of effect has been observed for other viruses. Data predicting HLA-specific binding of viral epitopes raise the hypothesis that HLA-A alleles have evolved primarily to modulate immune responses against DNA viruses, in contrast to HLA-B, which is better adapted for RNA viruses [6].

Determining the genetic determinants of antiviral immunity is key to informing our understanding of disease outcomes, and informing the development of immunotherapeutic strategies. We therefore set out to quantify the extent and nature of an HLA class I influence on HBV infection.

We investigated a composite cohort of human immunodeficiency virus (HIV)–infected adults from southern Africa, previously screened for HBV coinfection [7]. Our interest in this population is based on evidence that outcomes of both HBV and HIV infection are worse in the setting of coinfection and that there is an emerging burden of liver disease among HIV/HBV-coinfected subjects. These observations are of particular concern in settings where the two viruses are coendemic [8].

To test the hypothesis that HLA class I alleles are predictive of HBV virologic status, we sought evidence for any association between HLA class I and HBV phenotypes, based on hepatitis B surface antigen (HBsAg) status, hepatitis B e antigen (HBeAg) status, and HBV DNA load. Studying an HIV-positive cohort allowed us to compare the signals for HLA-mediated viremic control of HIV with those seen for HBV and to ascertain influences on HBV control in this particularly vulnerable population.

## METHODS

### Cohort Description and Ethics

We studied 1039 antiretroviral therapy–naive adults with chronic HIV infection from sub-Saharan Africa, who had previously been recruited through 4 cohorts (Table 1). The epidemiology of HBV/HIV coinfection in this region is further described by a recent study of cohorts from South Africa and Botswana [7], and by a recent review [8]. All participants provided written informed consent.

**Table 1.** Findings in 4 Cohorts of Antiretroviral Therapy–Naive HIV-Positive Adults From Southern Africa Investigated for the Presence of HBV Coinfection and for HIV/HBV Disease Markers

Finding	Cohort Location				
	Durban, South Africa (n = 425)	Kimberley, South Africa (n = 71)	Gaborone, Botswana (n = 380)	Thames Valley (n = 163)^a^	Total (n = 1039)
Recruitment site	Antenatal clinics	Mothers of HIV-infected children	Antenatal clinics	HIV outpatient clinic attenders	…
Ethics approval	University of KwaZulu-Natal Biomedical Research Ethics Committee, South Africa (reference E028/99)	Ethics Committee of the Faculty of Health Science, University of Free State, Bloemfontein, South Africa (reference ETOVS Nr 08/09)	Health Research and Development Division, Ministry of Health, Gaborone, Botswana (reference PPME-13/18/1)	University of Oxford Research Ethics Committee, UK (reference 06/Q1604/12)	…
Sex of cohort^b^	All female	All female	All female	Mixed male and female	…
HIV disease control, No. (% of subjects tested)^b,c^	50/413 (12.1)	2/25 (8.0)	75/375 (20.0)	53/150 (35.3)	180/963 (18.7)
Assay used for HBsAg testing	Biokit enzyme immune assay	Biokit enzyme immune assay	Murex HBsAg v3 assay	Roche COBAS, Biomerieux VIDAS, Abbott Architect	…
Assay used for HBeAg testing	Abbott Architect	ADVIA Centaur CP	ADVIA Centaur CP	ADVIA Centaur CP	…
HBsAg positive, No. (% of all subjects)	40/425 (9.4)	10/71 (14.1)	15/380 (3.9)	11/163 (6.7)	76/1039 (7.3)
HBeAg positive (% of subjects tested)^c^	12/40 (30.0)	2/8 (25.0)	2/9 (22.2)	2/8 (25.0)	18/65 (27.7)
Active HBV, No. (% of subjects tested)^c,d^	20/40 (50.0)	2/8 (25.0)	2/9 (22.2)	2/8 (25.0)	26/65 (40.0)

Abbreviations: HBeAg, hepatitis B e antigen; HBsAg, hepatitis B surface antigen; HBV, hepatitis B virus; HIV, human immunodeficiency virus.

^a^ The Thames Valley Cohort recruits adults attending HIV outpatient clinics operating from within hospital settings in the Thames Valley region of the United Kingdom. The patients represented in this study originate from a variety of countries in sub-Saharan Africa (>10 were recruited from 4 countries Zimbabwe [n = 77; 4 HBsAg positive], South Africa [n = 18; 1 HBV positive], Kenya [n = 12; none HBsAg positive], and Malawi [n = 11; 1 HBsAg positive]; 13 other countries were represented by <10 patients each).

^b^ HIV disease control is defined as HIV RNA <2000 copies/mL.

^c^ Owing to missing data or insufficient samples, the denominator for individual tests may be less than the total number of individuals in each cohort.

^d^ Active HBV is defined as HBeAg positive or HBV DNA >2000 IU/mL.

### Characterization of HBV Infection

HBsAg testing was undertaken, followed by additional testing of HBsAg-positive samples for HBeAg (subject to sample availability). These tests were performed with serum samples, using standardized assays according to the manufacturers' instructions (Table 1) [9]. HBV genotypes were ascertained from the HBV polymerase reverse-transcriptase domain, as described elsewhere [9], for 14 patients from Durban (9/14 genotype A1; 5/14 genotype A2) and 2 patients from Gaborone (both genotype D3), in whom DNA levels were sufficient for amplification.

We measured HBV DNA levels in subjects who were HBsAg positive but HBeAg negative (subject to availability of sample; n = 27), using real-time polymerase chain reaction (lower limit of detection for HBV DNA, 50 IU/mL), as described elsewhere [9]. In 8 of 27 subjects (29.6%), the HBV DNA level was >2000 IU/mL.

### Characterization of HIV Infection

We measured HIV-1 RNA loads from plasma using the Roche Amplicor Version 1.5 assay; this was available in 963 subjects (median load, 18 200 RNA copies/mL plasma; interquartile range, 3530–77 200 RNA copies/mL plasma). Of these subjects, 180 (18.7%) were defined as HIV-1 controllers (plasma HIV-1 load, ≤2000 copies/mL). We set this as the dependent variable in a regularized logistic regression analysis, as described elsewhere [10]. CD4^+^ T cells were quantified using flow cytometry in 989 subjects (median, 370 cells/mm^3^; interquartile range, 256–526 cells/mm^3^).

### HLA Typing

HLA class I type was determined for the Durban cohort by the South African Blood Bank Services and confirmed by the Carrington laboratory (Ragon Institute of MGH, MIT and Harvard). For other cohorts, HLA typing was performed by the Hildebrand laboratory (University of Oklahoma Health Sciences Center). HLA genotype was defined as the expression of a 2-digit HLA allele, with the exception of A*68 and B*15, which were resolved to 4 digits because they cross supertype boundaries. Alleles observed at ≥1% phenotypic frequency were included (total n = 51 [18 HLA-A, 21 HLA-B, and 12 HLA-C]; Supplementary Table 1).

### Statistical Analysis

We classified our patients according to 3 criteria: HBsAg status, HBeAg status; and “active” HBV disease (either HBeAg positive or HBV DNA >2000 IU/mL), using a computational algorithm similar to that which has been used in studies of HIV [11]. The contributions of HLA and cohort to HBsAg, HBeAg, and HIV controller status were estimated using elastic net regularized logistic regression [10]. Regularized logistic regression weights were optimized in a generalized least squares framework, with an added penalty for the size of the weights that serves to reduce the risk of overfitting. Overall model performance was evaluated using receiver operating characteristic curves evaluated on hold-out data from 10-fold cross-validation. For each train-test split, the elastic net λ parameter was chosen to be the one that maximized mean log likelihood on hold-out data, evaluated via nested 10-fold cross-validation on the training set, and α was set to 10%. Model weights (Supplementary Table 1) were trained on all the data, with the regularization parameter estimated via 10-fold cross-validation.

**Figure 1.**
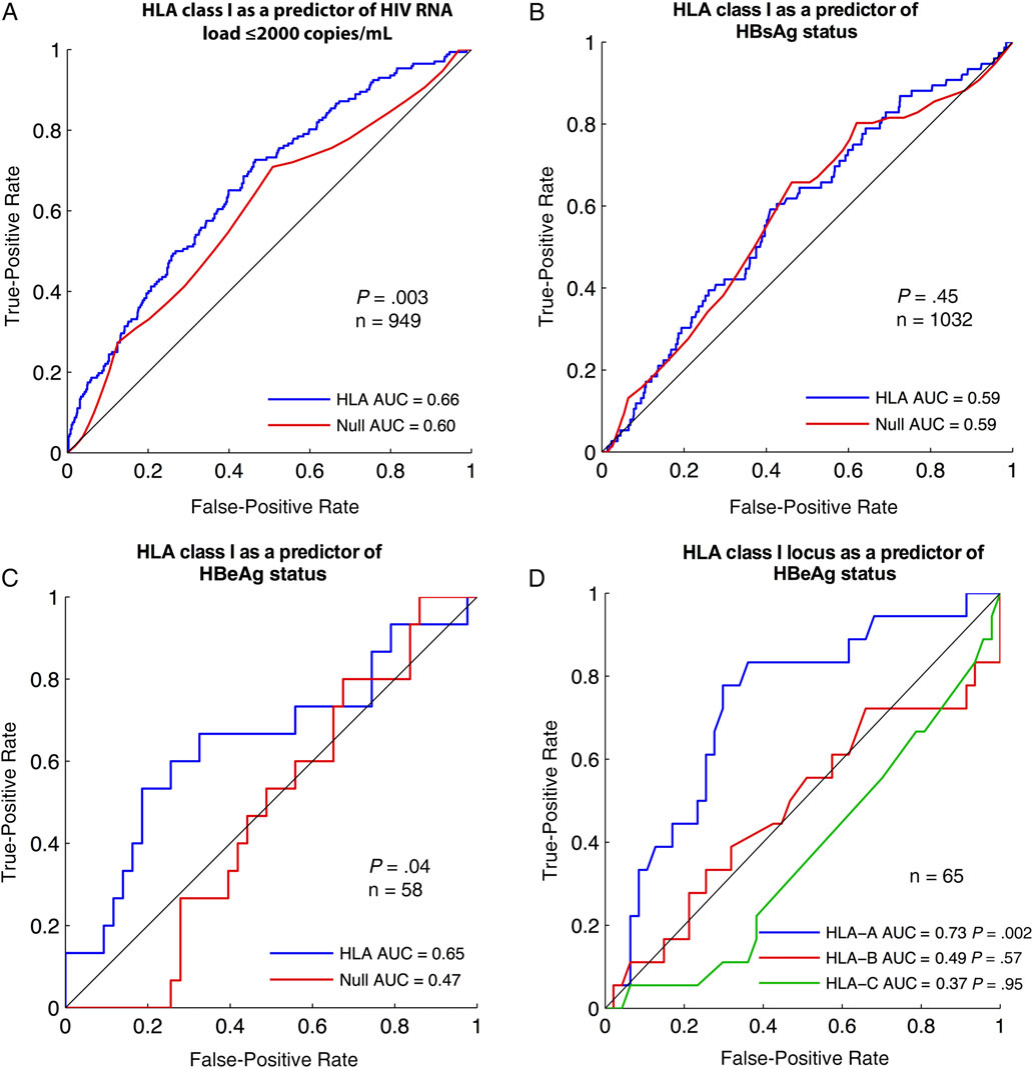
Receiver operating characteristic (ROC) curves for HLA class I as a predictor of disease status in chronic human immunodeficiency virus (HIV) and hepatitis B virus (HBV) infection. *A*, HLA class I as a predictor of HIV controller status (defined as plasma HIV-1 RNA load ≤2000 copies/mL). *B,* HLA class I as a predictor of hepatitis B surface antigen (HBsAg) status. *C,* HLA class I as a predictor of hepatitis B e antigen (HBeAg) status. In *A–C,* the red line represents analysis with cohort location only as predictor of virologic outcome; the blue line, a prediction incorporating HLA class I genotype plus cohort location. In *C,* HIV load and CD4 counts were also included in both models. *D*, HLA class I locus as a predictor of HBeAg status: HLA-A (*blue line*) versus HLA-B (*red line*) versus HLA-C (*green line*). The analysis in *D* does not correct for CD4^+^ T-cell count or HIV load, so 7 additional patients for whom these data were missing were included. Abbreviation: AUC, area under the curve.

We report the area under the curve (AUC) for models trained with various sets of features (Figure 1). For each analysis, we defined a null model consisting only of cohort location (as binary indicator variables; Table 1). For HBV severity end points (HBeAg positive or “active HBV”), the null models of the primary analyses also included HIV-1 RNA load (log_10_ transformed) and CD4^+^ T-cell counts (Box–Cox transformed). The alternative models also included HLA alleles. For all end points, the inclusion of HIV outcome markers (CD4^+^ T-cell count and HIV RNA load) did not significantly change model prediction.

The significance of receiver operating characteristic curves was estimated using a 1-sided Mann–Whitney *U* test to compare a single curve against random prediction, or via bootstrap (10 000 replicates) to test for a significant difference in AUC between 2 curves [12]. To test the theory that the HLA-A locus has the greatest impact on the control of DNA viruses [6], we built separate predictive models by HLA class I locus (Figure 1*D*). For these models, we excluded cohort labels and HIV clinical features because these provided no predictive value (Figure 1*C*); therefore, 7 additional subjects were added for whom HIV load and CD4^+^ data were missing.

## RESULTS

### Prevalence of HBsAg and HBeAg

Among this cohort of antiretroviral therapy–naive HIV-positive adults from southern Africa, 76 of 1039 (7.3%) were HBsAg positive. HBeAg status was determined for 65 of the 76 (86%), of whom 18 (28%) were HBeAg positive (Table 1).

### Impact of HLA Class I on HIV-1 and HBV Virologic Markers

We first sought to confirm the impact of HLA class I alleles on HIV disease control. As expected, HLA class I was a significant overall predictor of HIV-1 load (AUC, 0.66; *P* = .003; Figure 1*A*). Confirming this association is a helpful validation of our statistical approach to this cohort, because the result is concordant with previous findings (eg, [13]). HIV disease control did not differ between HBsAg-positive subjects (9 of 67) and HBsAg-negative subjects (171 of 896; *P* = .33, Fisher exact test). These numbers were too small to ascertain any HLA-specific influence on control within the HBsAg-positive group.

We next investigated for any influence of HLA class I on HBV markers. There was no relationship between HLA and HBsAg status (AUC, 0.59; blue line in Figure 1*B*). This effect was comparable to a model that used cohort location only as a predictor of HBsAg status (red line in Figure 1*B*). However, among HBsAg-positive subjects, HLA class I was a significant predictor of HBeAg status (*P* = .04; Figure 1*C*). The overall predictive value of HLA for HBeAg status was comparable to that observed for HIV controller status (AUC, 0.65 vs 0.66, respectively). Although plasma HIV-1 RNA load and CD4^+^ T-cell count have a possible relationship with HBeAg status, this was not statistically significant (Supplementary Figure 1), and these features did not alter the predictive capacity of the regularized regression models (HIV parameters did not significantly improve AUC in the full model [AUC, 0.65 vs 0.62; *P* = .22]).

### Locus-Specific Impact of HLA on HBeAg Status

It has been postulated that HLA-A is dominant in mediating control of DNA viruses [6]. Our model that used HLA-A alleles alone indeed predicted HBeAg status (AUC, 0.73; *P* = .002; Figure 1*D*), whereas there was no significant association with HLA-B or HLA-C alleles. This suggested that the effect demonstrated in Figure 1*C* was driven largely by gene expression at the HLA-A locus. HLA-A remained significant among the original set of 58 subjects, with cohort and HIV clinical features as covariates (AUC, 0.65; *P* = .04).

To investigate more broadly the effect of HLA-A on disease control, we repeated the analysis, this time seeking any relationship between HLA and our extended group of “active HBV.” Again, we found a significant relationship between HLA-A expression and active HBV (AUC, 0.7; *P* = .003; data not shown); the relationship was not significant for HLA-B or HLA-C.

## DISCUSSION

These data represent the first reported association between HLA class I and HBV virologic status, either defined by HBeAg status alone or based on the broader category of active HBV disease. Our findings support the view that the CD8^+^ T-cell immune response contributes to the immune control of HBV and suggest that this effect is predominantly driven by HLA-A restricted responses. This conclusion is consistent with a recent report documenting new CD8^+^ T-cell epitopes in HBV [3], a study demonstrating the presence of HLA escape mutations in HBV core protein [4], the modeling study that predicts a dominant role of the HLA-A locus in immune responses to DNA viruses [6], and a report that HLA-A*0301 is associated with HBV clearance [14].

However, our study has several limitations. Unfortunately, clinical and demographic data were not routinely collected for the majority of these patients, and we therefore cannot present a breakdown by age, sex, or other clinical diagnoses. The other most obvious caveat is low numbers, making it impossible to draw robust conclusions about the statistical impact of any individual allele on HBeAg status; (despite recruitment of >1100 subjects, only 7% of them were HBsAg positive, and 28% of this subgroup was HBeAg positive). However, estimated model weights provide hypotheses for future validation (Supplementary Table 1).

Despite uncertainty about the role of any single allele, a statistically robust signal has nevertheless emerged. This result undoubtedly warrants further investigation: future studies should consider recruitment of larger cohorts, replication in an HIV-negative population, and inclusion of different HBV genotypes.

Although we did not find a significant relationship between HIV-1 RNA load and HBeAg status, several previous studies of southern African populations have documented increased HBV replication markers among subjects with low CD4^+^ T-cell counts and high HIV-1 RNA load (reviewed in [8]). The direction of any possible effect is uncertain—does poorly controlled HIV predispose to higher rates of chronic HBV infection and increased HBV viremia, or is coinfection with HBV a cofactor in accelerating HIV disease progression? In this study, our observations remained statistically significant even after correction for HIV load, so this feature is not sufficient to explain the role of HLA class I in HBV control.

This study provides exciting new insights; the effect of HLA class I on HBeAg status is significant, with an AUC for HLA-A comparable in magnitude to that seen for HIV disease control. There have been increasing efforts to identify CD8^+^ T-cell epitopes in HBV proteins, including in e antigen [15], and to correlate these with disease outcome. Our data contribute to recent reports supporting the potential role for specific CD8^+^ T-cell responses in mediating control and clearance of HBV, thus informing the development of potential immunotherapeutic strategies to tackle HBV [5] as well as being potentially relevant to characterizing adaptive immune control of other viruses. These findings underpin the future development of therapeutic interventions that mimic the optimum responses observed in natural immune control of HBV.

## Supplementary Data

Supplementary materials are available at http://jid.oxfordjournals.org. Consisting of data provided by the author to benefit the reader, the posted materials are not copyedited and are the sole responsibility of the author, so questions or comments should be addressed to the author.

Supplementary Data
