# Supplementary material for: HLA-A is a Predictor of Hepatitis B e Antigen Status in HIV-Positive African Adults
Source: J Infect Dis. 2015 Dec 9;213(8):1248–52. doi: 10.1093/infdis/jiv592 (PMC4799671; doi:10.1093/infdis/jiv592)
Supplement: Supplementary Data [file supp_213_8_1248__index.html]

HLA-A is a Predictor of Hepatitis B e-Antigen Status in HIV-positive African Adults — HLA-A is a Predictor of Hepatitis B e Antigen Status in HIV-Positive African Adults — HLA-A is a Predictor of Hepatitis B e Antigen Status in HIV-Positive African Adults — Supplementary Data 

# HLA-A is a Predictor of Hepatitis B e Antigen Status in HIV-Positive African Adults

## Supplementary Data

Supplementary Data

- Supplementary Figure 1 - docx file
- Supplementary Table 1 - docx file
