# Supplementary material for: HLA-A is a Predictor of Hepatitis B e Antigen Status in HIV-Positive African Adults
Source: J Infect Dis. 2015 Dec 9;213(8):1248–52. doi: 10.1093/infdis/jiv592 (PMC4799671; doi:10.1093/infdis/jiv592)
Supplement: Supplementary Data [file supp_jiv592_jiv592supp_fig1.docx]

**
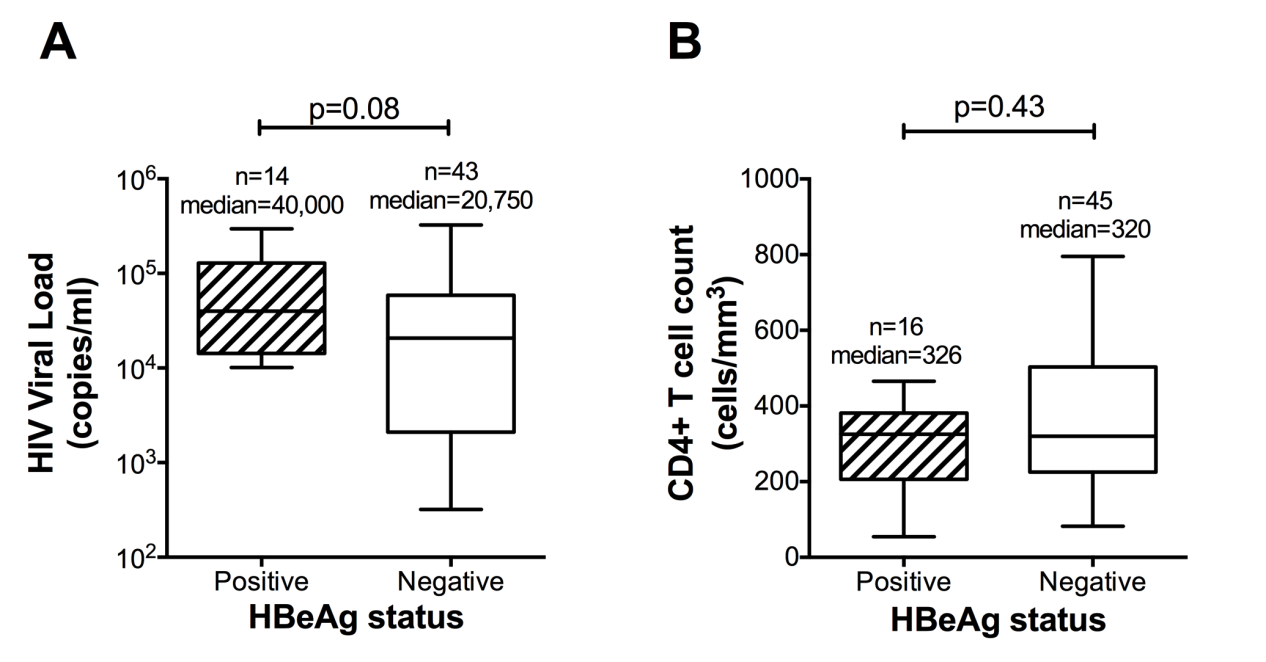
**

**Suppl. Fig. 1: Relationship between HBeAg status and HIV disease markers in a cohort of HIV/HBV co-infected African adults.** (A): HBeAg status vs. plasma HIV-1 RNA load (copies/ml) (B): HBeAg status vs. CD4+ T cell count (cells/mm^3^). P-values by Mann-Whitney U test. Boxes show median, 25^th^ and 75^th^ centiles, whiskers show 10^th^-90^th^ centiles. Number of individuals represented and median value is shown in each case.
