# Supplementary material for: HLA-A is a Predictor of Hepatitis B e Antigen Status in HIV-Positive African Adults
Source: J Infect Dis. 2015 Dec 9;213(8):1248–52. doi: 10.1093/infdis/jiv592 (PMC4799671; doi:10.1093/infdis/jiv592)
Supplement: Supplementary Data [file supp_jiv592_jiv592supp_table1.docx]

**Suppl. Table 1: Phenotypic frequency of HLA Class I alleles in a cohort of 1040 HIV-positive, ART-naïve, adults from southern Africa, and weight with respect to predicting HBeAg status among HBsAg-positive subset.** Alleles occurring at a phenotypic frequency of ≥1% (≥0.01) are represented. Weight is calculated for 65 HBsAg-positive patients with known HBeAg status using two different models (see footnote).

| **HLA-A allele** | **Phenotypic frequency^a^ (proportion)** | **Weight**  **(model A)^b^** | **Weight**  **(model B)^b^** |
| --- | --- | --- | --- |
| A*01 | 0.08 |  |  |
| A*02 | 0.25 | -0.23 | -0.35 |
| A*03 | 0.10 | 0 | -0.33 |
| A*23 | 0.18 | 0.56 | 0.85 |
| A*24 | 0.04 |  |  |
| A*26 | 0.03 |  |  |
| A*29 | 0.15 | 0 | 0.13 |
| A*30 | 0.39 | 0.32 | 0.99 |
| A*32 | 0.03 |  |  |
| A*33 | 0.05 | 0 | 0.03 |
| A*34 | 0.09 | -0.50 | -0.95 |
| A*36 | 0.02 |  |  |
| A*43 | 0.06 | 0 | 1.03 |
| A*66 | 0.07 | 0.91 | 1.56 |
| A*6801 | 0.07 | -0.76 | -0.82 |
| A*6802 | 0.15 | -0.50 | -0.72 |
| A*74 | 0.10 | 0.01 | -0.24 |
| A*80 | 0.02 |  |  |

| **HLA-B allele** | **Phenotypic frequency^a^**  **(proportion)** | **Weight**  **(model A)^b^** |
| --- | --- | --- |
| B*07 | 0.11 | -0.28 |
| B*08 | 0.10 | -0.29 |
| B*13 | 0.03 |  |
| B*14 | 0.08 |  |
| B*1501 | 0.01 |  |
| B*1503 | 0.16 | 0 |
| B*1510 | 0.14 | 0 |
| B*1516 | 0.01 |  |
| B*18 | 0.06 |  |
| B*35 | 0.06 |  |
| B*39 | 0.04 |  |
| B*41 | 0.02 |  |
| B*42 | 0.26 | 0 |
| B*44 | 0.16 | -0.31 |
| B*45 | 0.11 | -0.13 |
| B*49 | 0.01 |  |
| B*51 | 0.01 |  |
| B*53 | 0.07 |  |
| B*57 | 0.08 | 0 |
| B*58 | 0.28 | 0 |
| B*81 | 0.10 |  |

| **HLA-C allele** | **Phenotypic frequency^a^**  **(proportion)** | **Weight**  **(model A)^b^** |
| --- | --- | --- |
| C*02 | 0.19 | -0.26 |
| C*03 | 0.13 | 0 |
| C*04 | 0.26 | 0 |
| C*05 | 0.02 |  |
| C*06 | 0.24 | 0 |
| C*07 | 0.33 | 0.41 |
| C*08 | 0.12 | -0.49 |
| C*12 | 0.03 |  |
| C*15 | 0.02 |  |
| C*16 | 0.12 | 0 |
| C*17 | 0.28 | 0 |
| C*18 | 0.13 | -0.59 |

^a^ Phenotypic frequency was calculated by the on-line HLA Graphing tool at Los Alamos HIV databases: (<http://www.hiv.lanl.gov/content/immunology/hla/hla_graph.html>).

^b^ Weight values represent log-odds ratios with respect to HBeAg-positive status; positive values are associated with HBeAg-positive status and negative values with HBeAg-negative status. Weight is taken from the regularized logistic regression run, using a λ = 9.91 for the regularization parameter, as learned via 10-fold cross validation. For weight analysis, only alleles observed in ≥5 HBsAg+ individuals were considered. Alleles in red were excluded because of our mincount filter (i.e. there were <5 individuals with the allele out of the 65 with known HBeAg status).

Weight Model A was calculated using all Class I alleles as independent variables (as well as cohort and HIV load and CD4 count); Model B was derived from running just HLA-A alleles (on the basis of this locus standing out as significant in association with HBeAg status; with λ = 5.11). The discrepancies in weight calculations between models A and B for HLA-A reflect statistical uncertainty due to small numbers, differences in additional variables, and the corresponding changes in the regularization parameter. Model A corresponds to Fig 1C; Model B corresponds to Fig 1D.
